# Quantitative mapping of gut microbiota feature importance to TCM syndrome elements in clinical T2DM stages

**DOI:** 10.3389/fcimb.2026.1910757

**Published:** 2026-09-24

**Authors:** Ruifang Liu, Yingrong Liu, Jiawei Jiang, Zhaoyang Yang

**Affiliations:** 1 Rehabilitation College, Gannan Medical University, Ganzhou, Jiangxi, China; 2 TCM Syndrome Research Base, Fujian University of Traditional Chinese Medicine, Fuzhou, China

**Keywords:** fecal microbiota transplantation, gut microbiota, machine learning, Mendelian randomization, TCM syndrome elements, type 2 diabetes mellitus

## Abstract

**Introduction:**

The clinical phenotype of modern type 2 diabetes mellitus (T2DM) has diverged from the classical traditional Chinese medicine (TCM) syndrome of Xiao Ke (wasting-thirst), with obesity replacing emaciation as the dominant presentation and challenging traditional symptom-based syndrome differentiation. The TCM syndrome element system, a quantifiable framework inherently compatible with molecular-level parameters, positions the gut microbiota — causally linked to T2DM, stage-specific in composition, and amenable to targeted intervention — as an ideal molecular anchor for refining syndrome differentiation. However, the quantitative feature importance of specific gut microbes for individual syndrome elements remains undetermined.

**Methods:**

We enrolled 154 participants across three T2DM stages (51 pre-diabetes, 53 T2DM, 50 T2DM with complications) for 16S rDNA sequencing, syndrome element assessment, and machine learning.

**Results:**

Mendelian randomization identified 21 gut microbiota taxa and functional pathways causally implicated in T2DM in East Asian populations, providing biological priors for subsequent analyses. Support vector machine (SVM) models with SHAP values quantified microbial feature importance for individual syndrome elements, revealing progressive evolution from dampness, qi deficiency, and spleen (pre-diabetes) to dampness, phlegm, heat, and kidney (complications). *Bacteroides* and *Faecalibacterium* showed the highest feature importance for spleen (4.9% each), *Veillonella* showed the highest feature importance for dampness (3.4%), and *Bifidobacterium* was the top feature for phlegm (1.0%). Fecal microbiota transplantation (FMT) in 34 patients provided interventional evidence consistent with the model: 26.7% of post-FMT differentially abundant genera (4 of 15) and 7.5% of differentially abundant species (8 of 106) overlapped with top-ranking SVM features.

**Discussion:**

This proof-of-concept study provides the first quantitative mapping of gut microbiota feature importance to TCM syndrome elements, establishing an integrated framework combining MR-based causal prioritization, ML-based feature importance mapping, and FMT-based intervention validation for micro-syndrome differentiation.

## 1 Introduction

T2DM is a leading metabolic disorder worldwide, affecting approximately 11.2% of adults in China (Li et al., 2020; International Diabetes Federation, 2021; Sun et al., 2022). It is defined by progressive insulin resistance, relative insulin deficiency, and chronic metabolic dysregulation, driving the development of micro- and macrovascular complications (Brownlee, 2001; Alicic et al., 2017; Jia et al., 2025; American Diabetes Association, 2020). Despite advances in pharmacotherapy, T2DM remains a chronic, progressive condition; current interventions are therefore largely confined to glycemic control and complication prevention (Ahmad et al., 2022).

In Traditional Chinese Medicine, T2DM has historically been categorized as Xiao Ke(wasting-thirst), manifesting as polydipsia, polyphagia, polyuria, and weight loss, with pathogenesis attributed to yin deficiency and dryness-heat (Meng et al., 2023). However, contemporary T2DM patients are predominantly obese rather than emaciated, and the classic symptom tetrad is now observed in only a minority of cases. This phenotypic divergence underscores a fundamental limitation: traditional symptom-based syndrome differentiation lacks objective molecular correlates, hindering a comprehensive understanding of T2DM pathogenesis (Meng et al., 2023).

The gut microbiota is now recognized as a critical mediator of T2DM pathophysiology (Tilg and Moschen, 2014). Patients typically exhibit a reduction in butyrate-producing bacteria and an enrichment of opportunistic pathogens (Zhang et al., 2010; Qin et al., 2012; Karlsson et al., 2013; Zhang et al., 2013; Zhong et al., 2019; Wu et al., 2020), with distinct stage-specific shifts. For instance, pre-diabetic individuals show reduced Clostridiumand Akkermansia, whereas refractory T2DM is characterized by elevated Bacteroides vulgatusand Veillonella (Allin et al., 2018; Wang et al., 2022). Microbial metabolites, including short-chain fatty acids (SCFAs), bile acids, and branched-chain amino acids, mediate host–microbe crosstalk, modulating insulin sensitivity, energy homeostasis, and inflammatory tone (Koh et al., 2016; Wang et al., 2011; Thomas et al., 2009).

The TCM syndrome element system developed by Zhu Wenfeng provides a rigorous quantitative framework that decomposes clinical syndromes into disease location elements (e.g., spleen, kidney, liver) and disease nature elements (e.g., qi deficiency, dampness, phlegm, heat), each assigned a continuous severity score. Within this framework, the gut microbiome naturally extends the microscopic parameter dimension, providing molecular evidence to augment traditional four-diagnostic observations. SVM excel in high-dimensional, small-sample settings typical of microbiome studies (Cortes and Vapnik, 1995). Permutation importance quantifies model-level feature relevance by measuring performance degradation following feature permutation (Altmann et al., 2010), while SHAP (SHapley Additive exPlanations) values decompose predictions to offer both global and local interpretability (Lundberg and Lee, 2017; Lundberg et al., 2020). Nevertheless, systematic investigations applying machine learning to quantify the relationships between specific gut microbiota features and individual TCM syndrome elements across T2DM stages remain lacking.

FMT has demonstrated therapeutic potential for metabolic diseases, improving insulin sensitivity and glycemic control (Vrieze et al., 2012; Kootte et al., 2017; Mocanu et al., 2021; De Groot et al., 2017; Ng et al., 2022). Crucially, FMT provides a perturbation-based interventional model; comparing pre- and post-transplantation changes in microbiome composition and syndrome element scores allows us to validate the microbiota–syndrome element model within a causal framework. In this study, we integrated multi-omics profiling, TCM syndrome element quantification, Mendelian randomization, SVM-based machine learning, and FMT clinical validation. We aimed to quantitatively map the feature importance of specific gut microbiota to individual syndrome elements across T2DM progression. We hypothesized that distinct microbial taxa would exhibit unique feature importance profiles for specific syndrome elements, and that this pattern would be corroborated by both MR causal inference and FMT-induced clinical changes. Given the exploratory nature of this investigation and the multiplicity of analyses relative to our sample size, this study is principally designed as a proof-of-concept investigation.

## 2 Materials and methods

### 2.1 Study design and participants

This study comprised two complementary components: (1) a cross-sectional observational study of 154 participants across three T2DM stages for clinical characterization and syndrome element assessment, with gut microbiota sequencing performed on 201 participants from the same recruitment pool (comprising the 154 clinical participants and 47 additional individuals with available fecal samples) for microbiome analysis and machine learning; and (2) an FMT interventional validation cohort of 34 patients. The study was approved by the Ethics Committee of Fujian Second People’s Hospital (Approval No. SPHFJP-T2023006-02) and registered at the Chinese Clinical Trial Registry (Registration No. ChiCTR2300077946). All participants provided written informed consent.

#### 2.1.1 Cross-sectional cohort

Participants were recruited from the inpatient and outpatient departments of Fujian Second People’s Hospital between January and December 2024. They were stratified into three groups: pre-diabetes (Pre-DM, n = 51), T2DM (n = 53), and T2DM with complications (T2DM+Comp, n = 50). Diagnosis followed the 2020 Chinese Guidelines for T2DM Prevention and Treatment. Pre-diabetes was defined as fasting plasma glucose (FPG) of 5.6–6.9 mmol/L, and/or 2-h postprandial glucose of 7.8–11.0 mmol/L, and/or HbA1c of 5.7%–6.4%. T2DM was defined as FPG ≥ 7.0 mmol/L, and/or 2-h postprandial glucose ≥ 11.1 mmol/L, and/or HbA1c ≥ 6.5%. The T2DM+Comp group comprised patients with confirmed T2DM plus at least one complication, specifically diabetic retinopathy, nephropathy, peripheral neuropathy, peripheral vascular disease, gastrointestinal dysfunction, or diabetic foot.

Inclusion criteria included age 18–70 years, no antibiotic or probiotic use within one month prior to enrollment, and capacity to complete questionnaires and TCM four-diagnostic evaluations. Given the established impact of pharmacotherapies on gut microbiota, specific medication exclusions were implemented. α-glucosidase inhibitors (e.g., acarbose) were strictly excluded due to their direct mechanistic modulation of the intestinal environment. Use of GLP-1 receptor agonists within the preceding three months was also excluded based on reported microbiota-altering effects. Conversely, SGLT2 inhibitors and DPP-4 inhibitors were not excluded, as clinical evidence suggests these agents exert minimal alterations on fecal microbiota when used as add-on therapy to metformin. This approach was essential to mitigate selection bias, particularly in the T2DM+Comp group where such agents are recommended for cardiorenal protection. Additional exclusion criteria included type 1 diabetes, gestational diabetes, recent cardiovascular or cerebrovascular events (within six months), malignancy, chronic infections, psychiatric disorders, pregnancy or lactation, substance abuse, and eating disorders.

Antidiabetic medication use was documented for all participants. The Pre-DM group was predominantly treatment-naive (82%). The T2DM group included 64% using metformin, 23% using sulfonylureas, and 13% using DPP-4 inhibitors. The T2DM+Comp group had more complex regimens, including insulin use (40%). Mean disease duration was 4.2 ± 3.1 years in the T2DM group and 9.7 ± 5.3 years in the T2DM+Comp group.

#### 2.1.2 FMT validation cohort

The FMT cohort comprised 34 participants (20 T2DM, 14 T2DM with complications) who received oral microbiota capsules. Healthy donors (aged 18–40 years; BMI 18.5–28 kg/m²) underwent comprehensive screening, including health questionnaires covering medical history, family history, lifestyle factors, mental health, and infectious disease risk, with active smokers and alcohol consumers excluded. Mandatory stool and blood laboratory screening was conducted within four weeks before donation and repeated periodically. Qualified donors tested negative for enteric pathogens. Fecal microbiota were processed using an automated anaerobic extraction system, encapsulated under controlled conditions, and stored at −80 °C until administration (Xiamen Chengge Biotechnology Co., Ltd.). Donor–recipient pairing was guided by a machine learning-based model utilizing metagenomic sequencing data from the donor bank to select the optimal match. Each participant received 20 capsules per session, administered once every other day for three sessions, with each capsule containing over 2.5 × 10¹² CFU of viable bacteria. Clinical and microbiome assessments were performed at baseline and 12 weeks post-transvention. Throughout the study, participants maintained their habitual diets and stable hypoglycemic regimens, with strict prohibition of new antibiotic or probiotic use to control for treatment-related confounders. Notably, formal dietary assessment (e.g., food frequency questionnaires) was not conducted during the FMT phase.

### 2.2 TCM syndrome element assessment

Two board-certified TCM practitioners, each with over five years of clinical experience, independently collected four-diagnostic information using standardized Case Report Forms (CRF) aligned with the National Standards for TCM Clinical Diagnosis and Treatment Terminology. Discrepancies between assessors were resolved through consensus discussion. Clinical data integration followed a comprehensive diagnostic framework, incorporating macro-level factors (seasonal, climatic, and geographic influences), intermediate-level parameters (symptoms, signs, medical history, and psychological scales), and micro-level objective findings (laboratory tests, imaging, and pathological results).

Syndrome element scores were calculated using the Zhu Wenfeng Syndrome Differentiation System, which operationalizes the TCM Syndrome Element Diagnostic Scale. This system defines 11 disease location elements (e.g., heart, liver, spleen, lung, kidney, stomach, gallbladder, large intestine, bladder, uterus, and meridians) and 12 disease nature elements (e.g., qi deficiency, yang deficiency, yin deficiency, blood deficiency, essence deficiency, fluid deficiency, qi stagnation, blood stasis, phlegm, dampness, heat, and cold). Individual symptoms and signs were assigned weighted scores ranging from 0 to 4 (0 = absent, 1 = mild, 2 = moderate, 3 = severe, 4 = very severe). The composite score for each element was derived as the weighted sum of relevant items and normalized to a 0–300 scale. Severity grading was categorized as normal (<70), mild (70-100), moderate (100-150), or severe (≥150). For subsequent analyses, only syndrome elements with scores ≥70 were retained.

### 2.3 16S rDNA sequencing and bioinformatics

Fecal samples (3–5 g, mid-portion) were collected into preservation solution tubes and maintained at room temperature prior to processing. Genomic DNA was extracted using standardized laboratory protocols. DNA concentration and purity were assessed using a Multiskan™ GO microplate reader (Thermo Fisher Scientific), and integrity was verified via agarose gel electrophoresis. The V3–V4 hypervariable region of the 16S rRNA gene was amplified using the primer pair 341F (5′-CCTACGGGNGGCWGCAG-3′) and 806R (5′-GACTACHVGGGTATCTAATCC-3′). Amplicon libraries were prepared following the Illumina protocol. Individual libraries were quantified using a Qubit 3.0 fluorometer (Thermo Fisher Scientific). Libraries were then pooled at equimolar concentrations, with insert sizes validated using an Agilent 2100 Bioanalyzer and effective concentrations determined via quantitative PCR (qPCR). Sequencing was performed on an Illumina MiniSeq platform (Illumina, San Diego, CA, USA) by Xiamen Chengge Biotechnology Co., Ltd. (Xiamen, China).

Raw paired-end reads were merged and quality-filtered using FLASH v1.2.11 (Magoč and Salzberg, 2011). Chimeric sequences were identified and removed. Operational taxonomic units (OTUs) were clustered at 97% sequence similarity using QIIME v1.9.0 (Caporaso et al., 2010), and taxonomic classification was performed against the SILVA release 138 database. Alpha and beta diversity metrics were calculated within QIIME. Principal Coordinate Analysis (PCoA) was conducted based on UniFrac distance matrices to visualize community dissimilarities.

### 2.4 Mendelian randomization analysis

Two-sample MR was conducted to identify gut microbiota taxa and metabolic pathways with causal links to T2DM in East Asian and European populations (Davey Smith and Hemani, 2014). Given the current unavailability of large-scale GWAS data for specific TCM syndrome elements, the primary objective of this MR analysis was not to infer causality between microbiota and syndrome elements. Instead, MR served to identify microbial taxa with established causality for T2DM, thereby providing robust biological priors for feature selection and interpretation in downstream machine learning models.

Exposure data comprised gut microbiota GWAS summary statistics from three sources: the China National GeneBank DataBase (CNGBdb; 500 taxa, metagenomic sequencing, Chinese cohort), the MiBioGen consortium (196 taxa, 18,340 participants), and a Dutch GWAS cohort (412 taxa, 7,738 participants). Outcome data included T2DM from Pheweb Japan (45,383 cases, 132,032 controls) and FinnGen; diabetic peripheral neuropathy from FinnGen (1,419 cases, 195,047 controls); diabetic retinopathy from FinnGen (14,584 cases, 202,082 controls); and diabetic nephropathy (ebi-a-GCST90018832; 1,032 cases, 451,248 controls).

Instrumental variables (SNPs) associated with gut microbiota exposures were selected using significance thresholds of P < 1 × 10⁻^5^ for MiBioGen and Dutch GWAS data, and P < 5 × 10⁻^8^ for CNGBdb exposures and all outcome GWAS datasets. Linkage disequilibrium (LD) clumping was performed using the 1000 Genomes Project reference panel (EUR for European populations; EAS for East Asian populations) to ensure instrument independence (r² < 0.001; clumping window > 10,000 kb). Instrument strength was validated using the F-statistic, with only instruments exhibiting F > 10 retained for analysis; the mean F-statistic of retained instruments was reported. Harmonization of exposure and outcome datasets was performed to align effect alleles, incorporating allele frequency checks, resolution of palindromic SNPs via minor allele frequency inference, and removal of incompatible or strand-ambiguous variants.

The inverse-variance weighted (IVW) method served as the primary analytical approach. Sensitivity analyses included Cochran’s Q test for heterogeneity, the MR-Egger intercept test for horizontal pleiotropy, MR-PRESSO for outlier detection, and leave-one-out analysis to assess single-SNP influence. Multiple testing correction was applied across all microbial taxa using the Benjamini–Hochberg false discovery rate (FDR) procedure. All statistical analyses were conducted in R using the TwoSampleMR package.

### 2.5 Correlation analysis

Microbiota–syndrome element correlations were assessed using Pearson correlation (normally distributed variables) and Spearman rank correlation (non-normally distributed variables). Species with total relative abundance below 0.1% were excluded. The significance threshold was set at *P* < 0.05 with Benjamini–Hochberg FDR correction. Results were visualized as bubble plots with bubble size proportional to −log_10_(*P*) and color reflecting correlation direction and magnitude.

### 2.6 Machine learning model construction

#### 2.6.1 Feature matrix construction

The machine learning analysis utilized paired microbiota and syndrome element data from 201 participants with complete metagenomic sequencing and TCM syndrome evaluation. The input feature matrix Xcomprised genus-level (76 features) and species-level (287 features) relative abundances following low-abundance filtering (mean relative abundance > 0.01% and prevalence > 10% across samples). Compositional data were transformed using centered log-ratio (CLR) transformation and subsequently standardized using a StandardScaler(zero mean, unit variance). The target vector yencoded the severity grade for each syndrome element on a four-point ordinal scale (0: normal, 1: mild, 2: moderate, 3: severe). Separate supervised models were constructed for each syndrome element.

#### 2.6.2 SVM model training and optimization

Support Vector Machine (SVM) classifiers were implemented in Python (v3.9) using scikit-learn (v1.3.0). Initial filtering retained taxa with a mean relative abundance > 0.01% and prevalence > 10%. CLR transformation was applied to mitigate compositional constraints inherent in microbiome data, followed by standardization. Model performance was evaluated using a nested 10-fold cross-validation framework repeated five times. The outer loop assessed generalization performance, while the inner loop (5-fold GridSearchCV) optimized hyperparameters, including kernel type (linear, radial basis function), regularization parameter C(0.1, 1, 10, 100), gamma (scale, auto, 0.1, 0.01, 0.001), and class weights (balanced). This nested design prevented information leakage from hyperparameter tuning into performance estimation. Model performance was quantified using the area under the receiver operating characteristic curve (AUC), accuracy, macro-averaged F1-score, and additional ordinal-specific metrics (Quadratic Weighted Kappa and Spearman’s ρ; see Section 2.6.3), reported as mean ± standard deviation across the 50 outer-loop folds. Statistical significance was evaluated via permutation testing (99 label-randomization iterations) with Benjamini–Hochberg false discovery rate (FDR) correction applied across all syndrome element models; significance was defined as nominal P< 0.05 and FDR-corrected q< 0.05.

#### 2.6.3 Permutation importance and ordinal performance metrics

Model-level feature importance was quantified using permutation importance (Permutation Importance module, eli5 library; Altmann et al., 2010). For each fitted SVM model, feature columns were randomly permuted 30 times, and the mean decrease in AUC was recorded as the importance weight. Crucially, these importance weights reflect predictive relevance rather than direct biological causality. Weights were normalized as percentages within each model to facilitate cross-element comparisons. Features were ranked by descending importance and reported as relative percentages.

To address the ordinal nature of syndrome element severity, two complementary metrics were integrated alongside AUC. First, Quadratic Weighted Kappa (QWK) assessed agreement between predicted and true severity grades, applying squared penalties to larger discrepancies (Cohen, 1968). QWK ranges from −1 to 1, with values > 0 indicating performance exceeding chance. Second, Spearman’s rank correlation coefficient (ρ) was computed between the SVM decision function values (signed distance from the hyperplane) and the original continuous syndrome element scores (0–300 scale), quantifying monotonic relationships between predictions and clinical severity. Significance for these metrics was determined via permutation testing (99 shuffles; P< 0.05). All results are reported as mean ± standard deviation across the 50 outer-loop folds.

#### 2.6.4 SHAP value calculation

SHAP (SHapley Additive exPlanations) values were computed to provide instance-level interpretability of feature contributions. Summary plots and dependence plots were generated to visualize the directional impact of microbial features on syndrome elements. Positive SHAP values indicated contributions toward increased severity, whereas negative values indicated protective associations. These visualizations facilitated the identification of non-linear dose–response relationships between microbial abundance and clinical outcomes.

### 2.7 FMT validation cohort analysis

Analyses of the 34-patient FMT cohort were stratified by disease status (T2DM, n= 20; T2DM with complications, n= 14). Paired comparisons between baseline and week 12 post-intervention were conducted for: (1) biochemical parameters (HbA1c, 2-h postprandial glucose, FPG, BMI, waist/hip circumferences, visceral/subcutaneous fat, fasting/postprandial C-peptide); (2) TCM syndrome element scores; and (3) gut microbiota composition. Continuous variables were compared using the Wilcoxon signed-rank test, with Benjamini–Hochberg FDR correction applied across all comparisons; both nominal Pvalues and FDR-corrected qvalues are reported. Participant status was categorized as “diseased,” “pre-disease,” or “non-diseased” at each time point.

Validation of the ML-derived feature importance model was assessed through three complementary approaches: (1) quantification of overlap between post-FMT differentially abundant taxa and top-ranking SVM features; (2) evaluation of directional consistency between microbial abundance shifts and syndrome element score changes; and (3) comparative analysis of feature importance profiles and therapeutic response patterns between the T2DM and complication subgroups.

### 2.8 Statistical analysis

Group comparisons for continuous variables were performed using the Kruskal–Wallis test with Dunn’s *post-hoc* correction for non-normally distributed data (Shapiro–Wilk P> 0.05) and one-way ANOVA for normally distributed data. Categorical variables were compared using the chi-square test or Fisher’s exact test, as appropriate. Paired pre- and post-FMT analyses employed the Wilcoxon signed-rank test. Benjamini–Hochberg FDR correction was uniformly applied to all large-scale parallel tests, including: (i) differential abundance analyses across T2DM stages (**Figure 1D**); (ii) FMT pre- versus post-intervention abundance comparisons (**Supplementary Figure S2**); (iii) microbiota–syndrome element correlation analyses (**Figure 2**); (iv) Mendelian randomization analyses; and (v) permutation significance testing for SVM models; and (vi) syndrome element severity comparisons across the three T2DM stages (Kruskal–Wallis test with Dunn’s *post-hoc*; **Supplementary Table S2**). Both nominal Pvalues and FDR-corrected qvalues are reported throughout; statistical significance was defined as nominal P< 0.05 and FDR-corrected q< 0.05. All tests were two-tailed. Computational analyses were performed in Python (scikit-learn, eli5, SHAP, pandas, numpy, matplotlib, seaborn) and R (TwoSampleMR, vegan, phyloseq, ggplot2).

**Figure 1:**
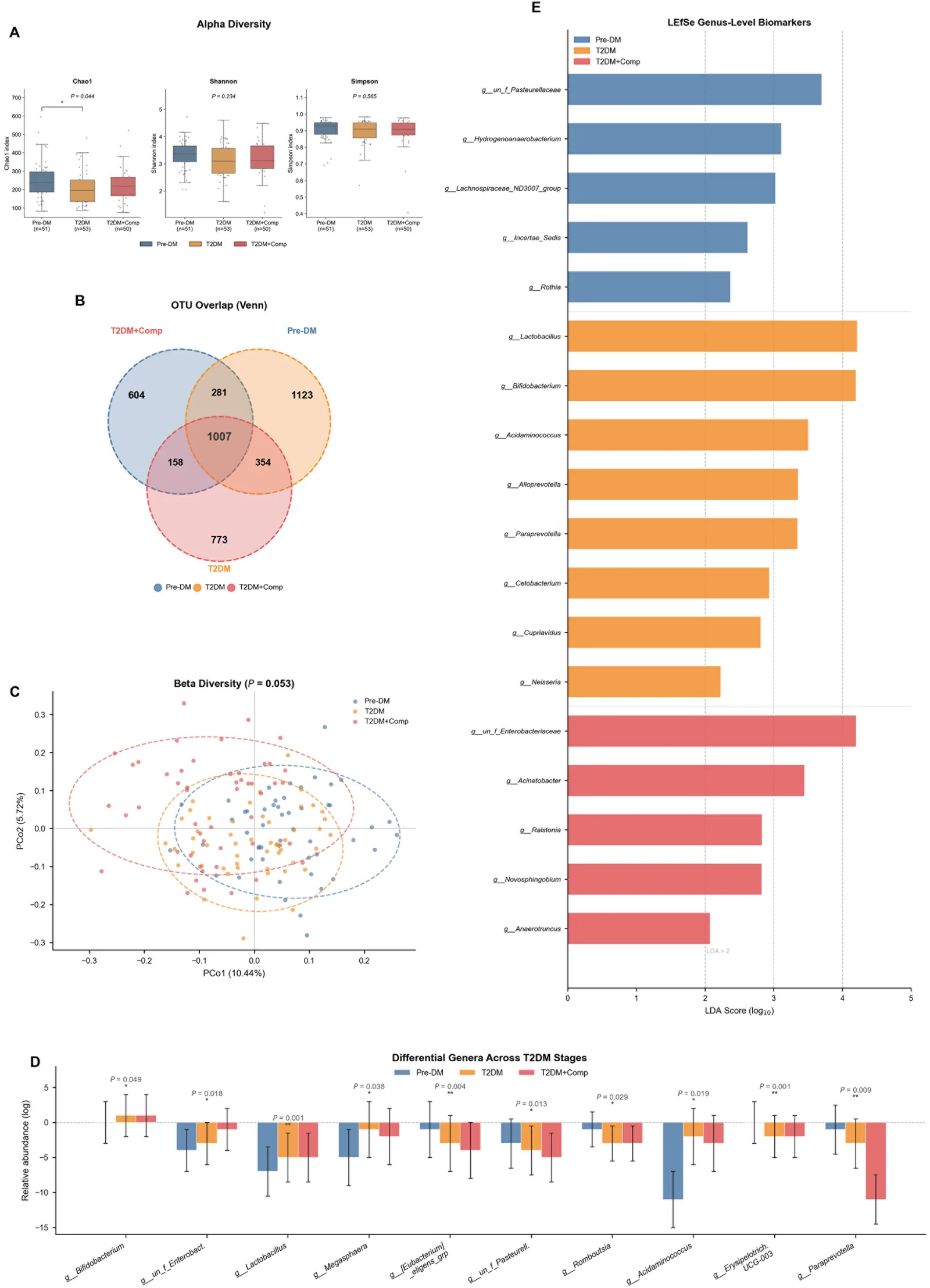
Gut microbiota characteristics across T2DM stages. **(A)** Alpha diversity indices (Chao1, Shannon, and Simpson) were compared across the three groups using the Kruskal-Wallis test with Dunn’s *post-hoc*. **(B)** Venn diagram of shared and unique OTUs. **(C)** PCoA based on UniFrac distances (PCo1 = 10.44%, PCo2 = 5.72%); ellipses, 95% CI. ANOSIM: R = 0.046, *P* = 0.014. **(D)** Differentially abundant genera (CLR-transformed relative abundance, mean ± SD; Kruskal-Wallis test with Benjamini–Hochberg FDR correction). **(E)** LEfSe-identified genus-level biomarkers (LDA > 2.0). Groups: Pre-DM (n = 51), T2DM (n = 53), T2DM+Comp (n = 50).

**Figure 2:**
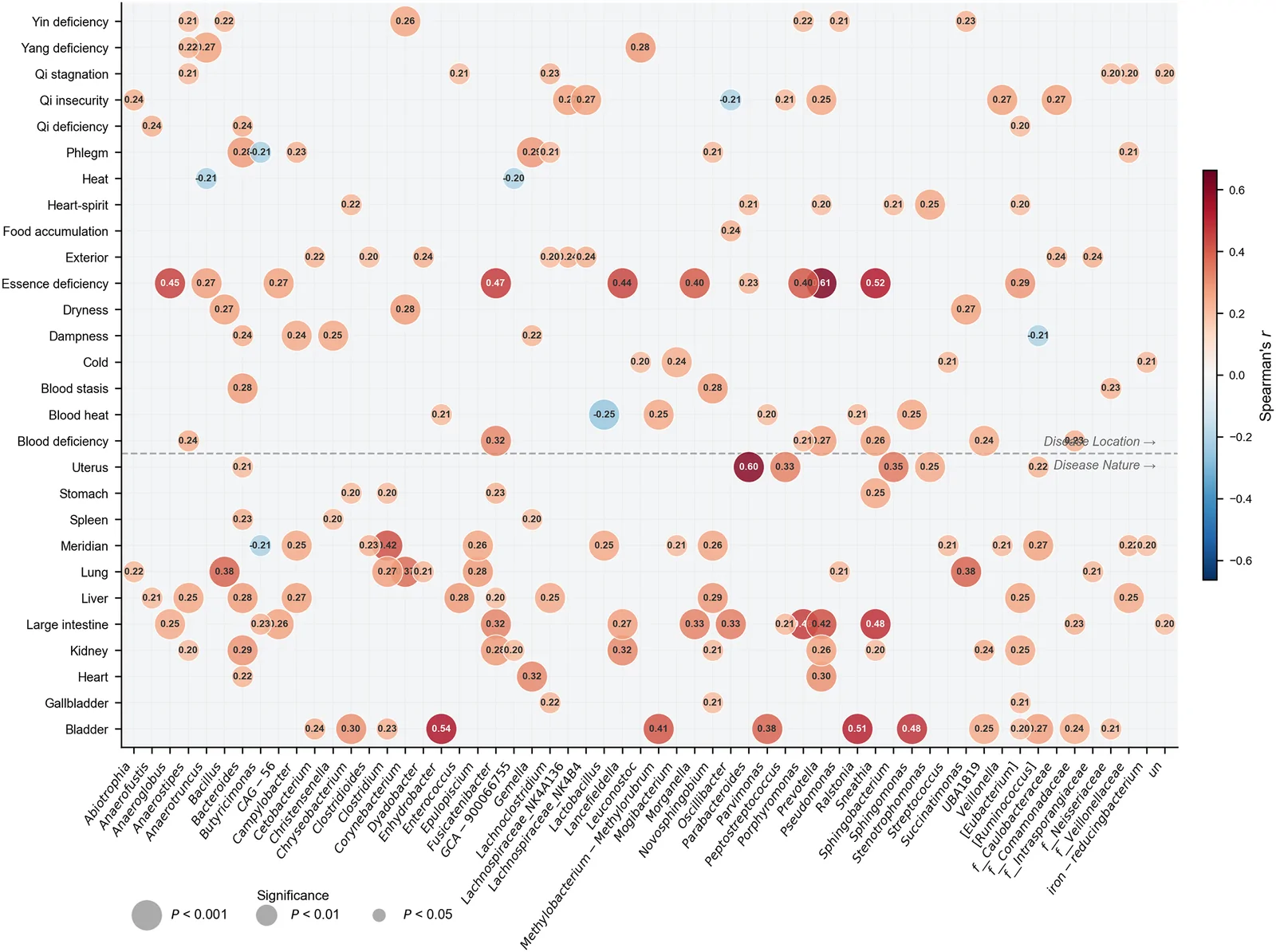
Correlations between gut microbiota genera and TCM syndrome elements. Bubble heatmap of Spearman correlation coefficients (|r| ≥ 0.2, FDR-corrected; n = 176 significant pairs shown). Color intensity indicates correlation strength and direction (red, positive; blue, negative). Bubble size reflects significance level (*P* < 0.001, *P* < 0.01, or *P* < 0.05). Syndrome elements are categorized into Disease Location (above dashed line) and Disease Nature (below dashed line). Numerical labels denote Spearman’s r values for each significant association.

## 3 Results

### 3.1 Baseline characteristics

The three groups were comparable in age, BMI, blood pressure, waist circumference, and most biochemical parameters (**Table 1**). Significant differences were observed in glycemic indices: HbA1c (Pre-DM 6.08 ± 0.43% vs. T2DM 8.00 ± 1.31% vs. T2DM+Comp 8.49 ± 1.86%, *P* < 0.001), FPG (Pre-DM 5.77 ± 0.92 vs. T2DM 8.84 ± 2.60 vs. T2DM+Comp 9.38 ± 3.31 mmol/L, *P* < 0.001), and 2 h postprandial glucose (Pre-DM 6.93 ± 1.91 vs. T2DM 12.91 ± 5.20 vs. T2DM+Comp 13.43 ± 4.78 mmol/L, *P* < 0.001). Waist-to-hip ratio differed between Pre-DM and T2DM+Comp groups (0.86 ± 0.06 vs. 0.91 ± 0.07, *P* = 0.048). Serum magnesium levels were also significantly different (*P* = 0.023), with lower values in the T2DM and T2DM+Comp groups.

**Table 1:** Baseline characteristics of study participants (n = 154).

Characteristic	Pre-DM (n = 51)	T2DM (n = 53)	T2DM+Comp (n = 50)	P-value
Demographics & anthropometrics				
Age (years)	54 (46, 65)	52 (46, 62)	53 (49, 60)	0.855
BMI (kg/m2)	25.6 (22.9, 27.8)	25.5 (23.2, 27.9)	25.3 (23.5, 27.5)	0.920
Waist-to-hip ratio	0.86 (0.80, 0.90)	0.90 (0.90, 0.90)	0.91 (0.90, 1.00)	0.048
Glycemic control				
HbA1c (%)	6.08 (5.88, 6.33)	8.00 (6.88, 8.75)	8.49 (6.85, 10.60)	<0.001
FPG (mmol/L)	5.77 (5.22, 6.17)	8.84 (7.32, 10.26)	9.38 (7.03, 11.63)	<0.001
2hPG (mmol/L)	6.93 (5.59, 7.92)	12.91 (8.32, 16.14)	13.43 (10.81, 17.36)	<0.001
Lipid profile				
Triglycerides (mmol/L)	1.53 (1.08, 2.14)	1.68 (1.12, 2.41)	1.73 (1.17, 2.85)	0.864
Total cholesterol (mmol/L)	5.46 ± 1.07	5.47 ± 1.26	5.29 ± 1.07	0.868
Other laboratory				
Serum magnesium (mmol/L)	0.83 (0.78, 0.88)	0.78 (0.72, 0.84)	0.78 (0.72, 0.82)	0.023
Clinical history				
Disease duration (years)	—	4.2 ± 3.1	9.7 ± 5.3	<0.001
Antidiabetic medications, n (%)				
Metformin	0 (0%)	36 (68%)	28 (56%)	<0.001
Insulin	0 (0%)	3 (6%)	20 (40%)	<0.001
GLP-1 RA	0 (0%)	2 (4%)	5 (10%)	0.052
DPP-4i	0 (0%)	7 (13%)	6 (12%)	0.029
Sulfonylureas	0 (0%)	12 (23%)	8 (16%)	0.002
Treatment-naive	42 (82%)	8 (15%)	0 (0%)	<0.001
Complications, n (%)				
Diabetic peripheral neuropathy (DPN)	0 (0%)	0 (0%)	26 (52%)	<0.001
Diabetic nephropathy (DN)	0 (0%)	0 (0%)	15 (30%)	<0.001
Diabetic retinopathy (DR)	0 (0%)	0 (0%)	13 (26%)	<0.001
Hypertension (HTN)	0 (0%)	0 (0%)	29 (58%)	<0.001
Coronary heart disease (CHD)	0 (0%)	0 (0%)	11 (22%)	<0.001
Cerebrovascular disease (CeVD)	0 (0%)	0 (0%)	6 (12%)	<0.001
Diabetic foot (DF)	0 (0%)	0 (0%)	4 (8%)	<0.001
Peripheral arterial disease (PAD)	0 (0%)	0 (0%)	7 (14%)	<0.001

### 3.2 Gut microbiota characteristics across T2DM stages

Alpha diversity analysis revealed significant differences in the Chao1 estimator across groups (*P* = 0.044), with species richness declining from pre-diabetes to complications. Only 1,007 OTUs were shared among all three groups, while unique OTU counts decreased with disease severity: 1,123 (Pre-DM), 773 (T2DM), and 604 (T2DM+Comp) (**Figure 1**).

PCoA based on UniFrac distances revealed modest between-group separation (ANOSIM R = 0.046, *P* = 0.014), indicating that group membership explains approximately 4.6% of the variation in microbial community structure. While the separation reached statistical significance, the small R value reflects substantial overlap among groups, and this trend should be interpreted with caution. The T2DM group showed the greatest dispersion along PC1 (10.44% variance) and PC2 (5.72% variance).

At the genus level, *g:Bacteroides* and *g:Prevotella* were dominant across all groups. Significantly differentially abundant genera (Kruskal–Wallis test with Benjamini–Hochberg FDR correction, q < 0.05) included *g:Bifidobacterium* (*P* = 0.049), *g:un_f_Pasteurellaceae* (*P* = 0.013), *g:un_f_Enterobacteriaceae* (*P* = 0.018), *g:Romboutsia* (*P* = 0.029), *g:Lactobacillus* (*P* = 0.001), *g:Acidaminococcus* (*P* = 0.019), *g:Megasphaera* (*P* = 0.038), *g:Erysipelotrichaceae_UCG-003* (*P* = 0.001), g:[Eubacterium]_eligens_group (*P* = 0.004), and g:Paraprevotella (*P* = 0.009).

LEfSe analysis identified distinct biomarker taxa for each group: the T2DM group was enriched in Firmicutes members including *Lactobacillus*, *Bifidobacterium*, and *Clostridiales*; the T2DM+Comp group was characterized by *Methylobacterium*, *Novosphingobium*, and *Megasphaera* enrichment; the Pre-DM group showed elevated *Erysipelotrichaceae*, *Eubacterium*, and *Bacteroides*. KEGG pathway enrichment revealed significant enrichment of the MAPK signaling pathway and chemical carcinogenesis–receptor activation pathway.

### 3.3 Syndrome element distribution across T2DM stages

Analysis of TCM syndrome elements in the 154 enrolled participants (51 pre-diabetes, 53 T2DM, 50 T2DM with complications) revealed a distinct stepwise evolutionary pattern across disease stages (**Figure 3**). In the pre-diabetes group, the predominant syndrome elements were dampness, qi deficiency, yang deficiency, and spleen, with most cases presenting at mild-to-moderate severity. The T2DM group exhibited a broadened clinical profile, retaining the core elements of dampness, spleen, yang deficiency, and qi deficiency while adding phlegm as a novel feature; the proportion of moderate-to-severe presentations increased substantially compared with the pre-diabetes cohort. The T2DM with complications subgroup displayed the most heterogeneous phenotype, encompassing dampness, phlegm, spleen, qi deficiency, heat, yang deficiency, and kidney. Notably, heat and kidney were uniquely observed in this subgroup, with severity grades shifting predominantly to the moderate-to-severe range.

**Figure 3:**
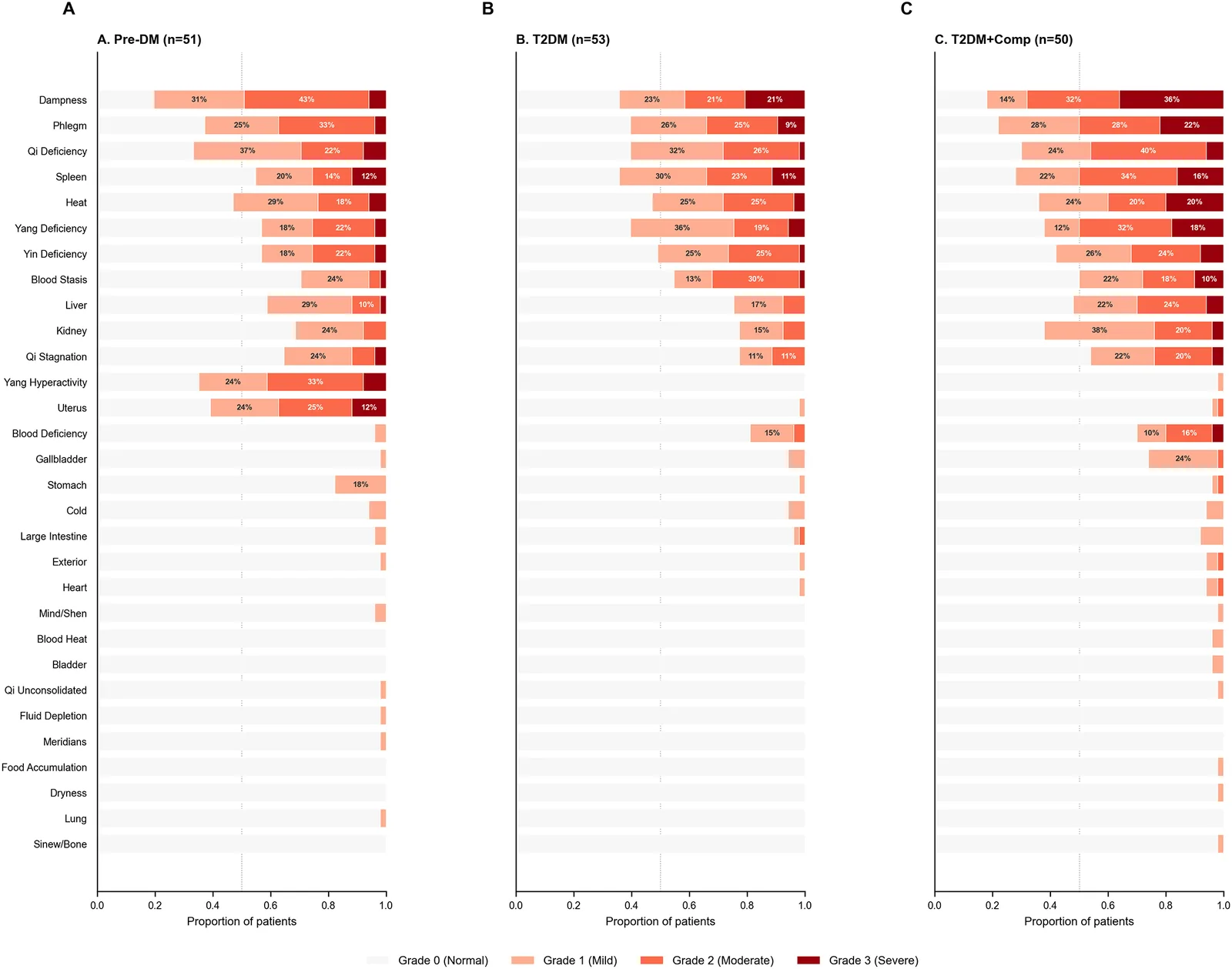
TCM syndrome element distribution across T2DM stages. Stacked horizontal bar charts showing the proportion of patients with each syndrome element graded by severity (Grade 0, normal; Grade 1, mild; Grade 2, moderate; Grade 3, severe). **(A)** Pre-DM (n = 51). **(B)** T2DM (n = 53). **(C)** T2DM+Comp (n = 50).

This stepwise progression aligns with the well-established clinical trajectory of T2DM, spanning early metabolic dysregulation to progressive multi-organ involvement. Specifically, the phenotype evolved from the dampness–qi deficiency–spleen signature characteristic of pre-diabetes, to the expanded dampness–phlegm–spleen profile in established T2DM, and ultimately to the multifaceted dampness–phlegm–heat–kidney phenotype in advanced complicated disease.

To formally evaluate whether syndrome element severity differed significantly across the three disease stages, Kruskal–Wallis tests were performed for each of the 51 syndrome elements, with Dunn’s *post-hoc* pairwise comparisons and Benjamini–Hochberg false discovery rate (FDR) correction. Effect sizes were quantified using epsilon-squared (ϵ²) for omnibus tests and Cliff’s delta (δ) for pairwise contrasts, where δ values of 0.11–0.28, 0.28–0.43, and >0.43 denote small, medium, and large effects, respectively.

The Kidney (Shen) element exhibited the most pronounced between-group difference (H = 8.63, *P* = 0.013, ϵ² = 0.045), with *post-hoc* analyses revealing a significant severity gradient: participants with T2DM and complications showed higher Kidney deficiency grades than both pre-diabetes individuals (Cliff’s δ = −0.354, P = 0.004, medium effect) and those with uncomplicated T2DM (Cliff’s δ = −0.327, P = 0.020, small-to-medium effect). The Gallbladder (Dan) element also reached nominal significance (H = 6.91, P = 0.032, ϵ² = 0.036), albeit with a smaller pairwise effect (Pre-DM vs. T2DM+Comp: δ = −0.217, P = 0.012). After FDR correction across all 153 comparisons (51 elements × 3 pairwise contrasts), no element retained q < 0.05—a finding consistent with the limited statistical power inherent to sparse ordinal data, where most syndrome elements had fewer than 15% of participants with non-zero grades. Nevertheless, the Kidney signal remained the most robust across multiple independent analytical approaches, converging with feature importance and correlation findings (Sections 3.5–3.6). Complete statistical results for all 51 syndrome elements are provided in **Supplementary Table S2**.

### 3.4 Mendelian randomization causal inference

To identify microbiota with causal evidence for T2DM that could serve as biologically informed candidates for microbiota–syndrome element analyses, Mendelian randomization was performed in East Asian and European populations. In East Asian populations (CNGBdb), 21 gut microbiota taxa and pathways were causally associated with T2DM (IVW method, *P* < 0.05): 14 were protective (MF0098, MF0067, MF0063, MF0011, *s:Prevotella_oris*, MF0060, *o:Erysipelotrichales*, *s:Butyrivibrio_crossotus*, *f:Streptococcaceae*, *s:Marvinbryantia_formatexigens*, *s:[Clostridium]_bartlettii*, *s:Campylobacter_jejuni*, *s:Turicibacter_sanguinis*, *s:Bifidobacterium_catenulatum*) and 7 were risk-associated (*f:Moraxellaceae*, *s:Filifactor_alocis*, *c:Betaproteobacteria*, *s:Brucella_melitensis*, *s:Streptococcus_gordonii*, *s:Ruminococcus_champanellensis*, and MF0049).

In European populations (Dutch GWAS, FinnGen), 8 microbiota–pathway associations were identified. Risk factors included *g:Prevotella* (OR = 1.08, 95% CI 1.02–1.15, *P* = 0.010), *g:Anaerotruncus* (OR = 1.03, 95% CI 1.00–1.07, *P* = 0.039), *s:Streptococcus_parasanguinis* (OR = 1.04, 95% CI 1.00–1.09, *P* = 0.033), and the PANTO.PWY pathway (OR = 1.06, 95% CI 1.01–1.12, *P* = 0.014). Protective factors included PWY.5667 (OR = 0.93, 95% CI 0.89–0.97, *P* = 0.002), PWY.6892 (OR = 0.93, 95% CI 0.87–0.99, *P* = 0.029), PWY.7221 (OR = 0.91, 95% CI 0.86–0.96, *P* = 0.001), and *s:Paraprevotella_clara* (OR = 0.96, 95% CI 0.94–0.99, *P* = 0.016).

For diabetic complications, MR identified 8 microbiota associated with peripheral neuropathy, 4 with retinopathy, and 12 with nephropathy; detailed taxa are listed in **Figure 4**. All MR results passed sensitivity analyses: Cochran’s Q test showed no significant heterogeneity, the MR-Egger intercept test detected no horizontal pleiotropy, MR-PRESSO confirmed robustness, and leave-one-out analysis demonstrated that no single SNP drove the causal estimates.

**Figure 4:**
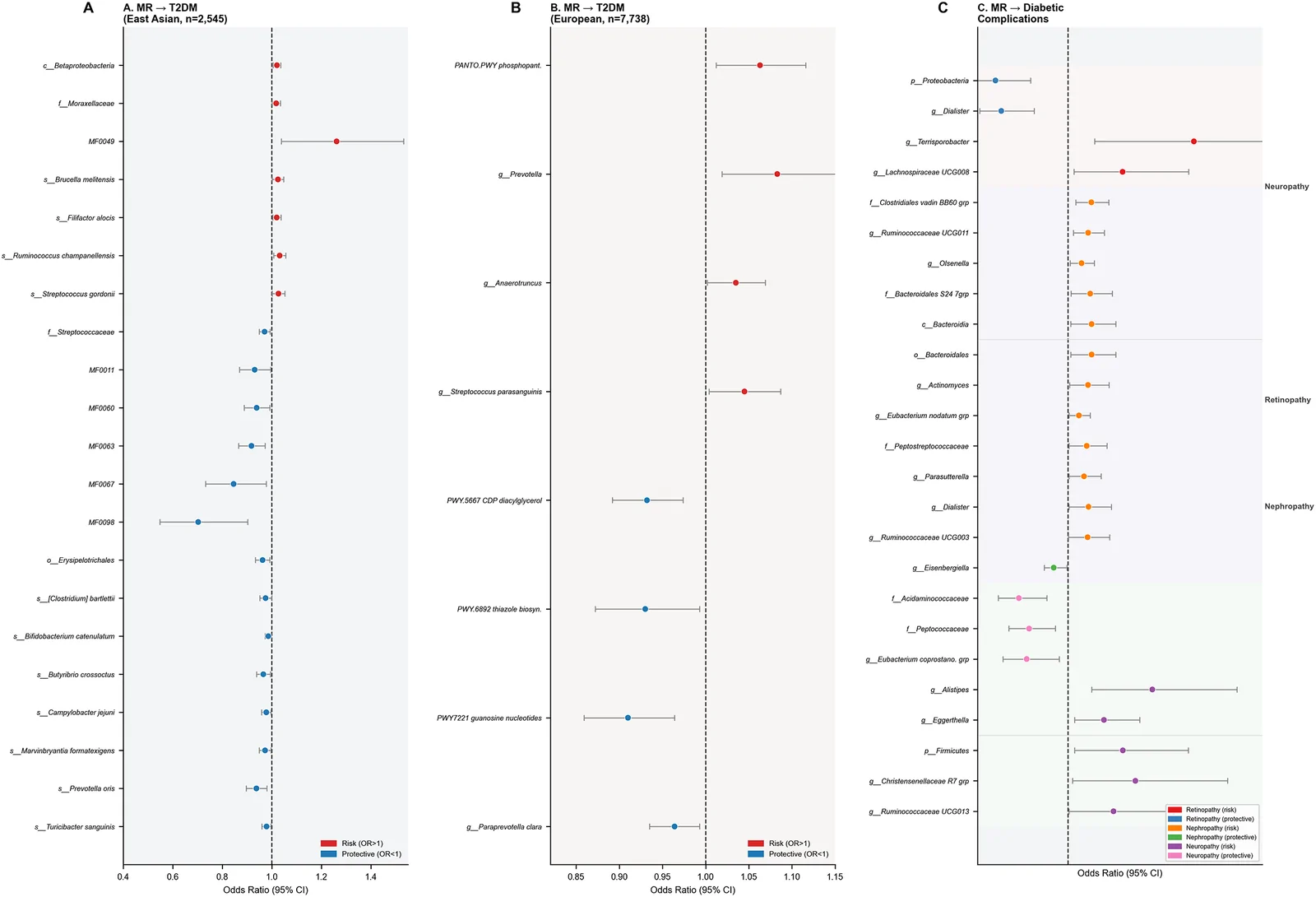
Causal effects of gut microbiota genera on T2DM and its complications from Mendelian randomization analysis. Forest plots showing odds ratios (OR) with 95% confidence intervals derived from inverse-variance weighted (IVW) estimation. **(A)** Causal estimates for T2DM (East Asian populations, 21 gut microbiota taxa and pathways). **(B)** Causal estimates for T2DM (European populations, 8 microbial features). **(C)** Causal estimates for diabetic complications; genera are colored by phylum and stratified into biomarker and replication sets. OR > 1 indicates risk effect; OR < 1 indicates protective effect.

### 3.5 Microbiota–syndrome element correlations

Multiple strong correlations (|r| ≥ 0.5, *P* < 1e-13) were identified between specific taxa and syndrome elements (**Figure 2**; complete data in **Supplementary Table S3**).

Several species were associated with multiple syndrome elements: *s:Sneathia_amnii* showed strong positive correlations with both large intestine and essence deficiency, suggesting convergent pathological mechanisms (**Table 2**). Most significant correlations were positive, indicating that higher abundance of specific taxa was associated with greater syndrome element severity, consistent with a pathogenic role for these microbes in TCM pathophysiology.

**Table 2:** Top microbiota–syndrome element correlations (species level, |r| ≥ 0.5).

Species	Syndrome element	r	*P*-value
s__un_g_Hafnia-besumbacterium	Uterus	0.648	4.86×10⁻²³
s__Prevotella_multisaccharivorax	Essence deficiency	0.613	3.85×10⁻^20^
s__Parabacteroides_distasonis	Large intestine	0.604	1.87×10⁻¹^9^
s__Sneathia_amnii	Essence deficiency	0.524	3.07×10⁻¹^4^
s__un_g_Pseudomonas	Bladder	0.511	1.66×10⁻¹³

### 3.6 Feature importance analysis

We trained SVM models for each syndrome element using a nested cross-validation framework (10-fold, repeated 5 times) with 99-permutation testing to derive empirical *P*-values. Thirteen elements met the inclusion criterion of having at least three samples in the rarest severity grade. Model performance was benchmarked against eight complementary metrics: AUC, accuracy, macro-averaged F1-score, balanced accuracy, Cohen’s κ, Quadratic Weighted Kappa (QWK), Spearman’s ρ (correlating decision function values with raw syndrome scores), and Top-2 accuracy. Full performance matrices are provided in **Supplementary Table S1**.

Genus-level AUCs clustered near chance (0.46–0.76), with Exterior yielding the highest value. However, conventional AUC proved insensitive to ordinal structure. In contrast, QWK—suited for ranked outcomes—detected significant signal where AUC did not. At the genus level, QWK identified ordinal structure for qi deficiency (0.08, *P* = 0.02), phlegm (0.09, *P* = 0.02), and yin deficiency (0.09, *P* = 0.04). At the species level, QWK additionally captured signal for heat (*P* = 0.01) and blood stasis (*P* = 0.03). Cumulatively, QWK identified significant structure in 6 of 13 species-level models compared to only 2 of 13 for AUC, indicating that ordinal metrics better exploit the information density of microbiome data in this context.

Kidney (Shen) was the most robust element across both taxonomic levels. At the genus level, it yielded an AUC of 0.55 ± 0.11 (*P* = 0.04), QWK of 0.08 (*P* = 0.04), and ρ = 0.18 ± 0.20 (*P* = 0.02). At the species level, AUC was 0.61 ± 0.10 (*P* = 0.01), QWK 0.09 (*P* = 0.03), and ρ = 0.23 ± 0.20 (*P* = 0.01). The consistent positive ρ across levels suggests a weak but reproducible monotonic relationship between microbial profiles and renal deficiency severity. Notably, Kidney was the only element to retain significance in Spearman correlation at both genus and species levels.

The Exterior element, representing superficial TCM pathogen invasion, exhibited the highest AUC (genus: 0.76, *P* = 0.04; species: 0.83, *P* = 0.01) and QWK (0.25, *P* = 0.01). However, this finding is tempered by extreme class imbalance (8 positives in 193 samples), limiting clinical generalizability. Species-level performance otherwise mirrored genus-level trends (AUC range: 0.44–0.83), though absolute performance remained modest (median AUC 0.49). Despite this, we observed phylogenetic conservation of feature importance; for instance, Bacteroides, Faecalibacterium, and Alistipesspecies consistently ranked highly for spleen across both taxonomic levels. Divergence between genus and species rankings likely reflects intra-genus functional heterogeneity—a known limitation of 16S rRNA profiling.

#### 3.6.1 Genus-level feature importance

Permutation importance revealed distinct microbial signatures for each syndrome element (**Figure 5A**, **Table 3**):

**Figure 5:**
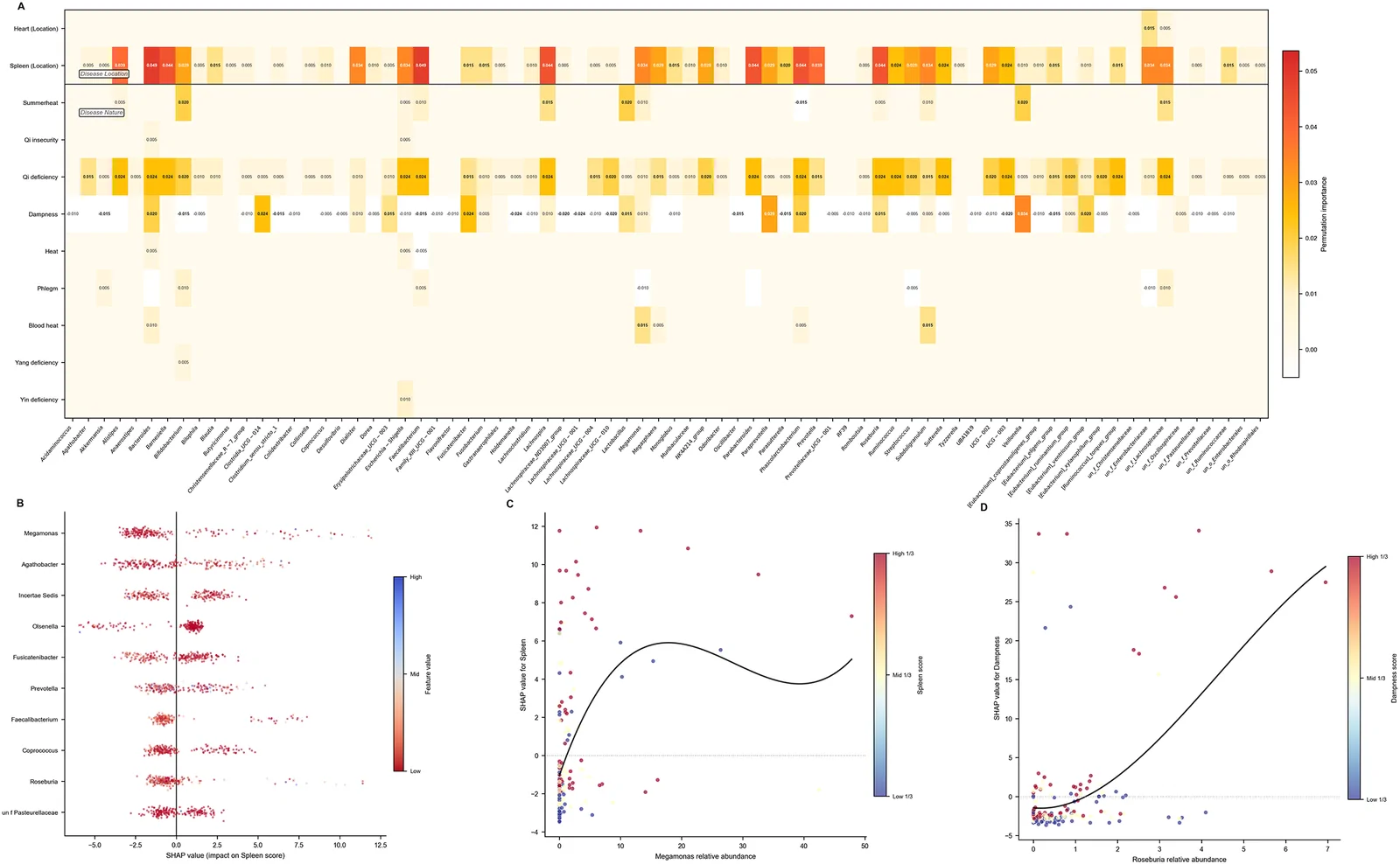
Machine learning identification of key microbiota feature importance profiles of TCM syndrome elements. **(A)** Permutation importance heatmap (76 genera × 11 syndrome elements); color intensity reflects feature importance feature importance magnitude, with nominal P values labeled. **(B)** SHAP beeswarm plot for Spleen (top 10 genera by mean |SHAP|). **(C)** SHAP dependence plot showing non-linear effect of Bacteroides on Spleen. **(D)** SHAP dependence plot showing positive linear association between Roseburia abundance and Dampness.

**Table 3:** Top ranked gut microbiota by feature importance for TCM syndrome elements.

Syndrome element	Top 1 genus	Feature importance (%)	Top 2 genus	Feature importance (%)	Top 3 genus	Feature importance (%)
Spleen	g__Bacteroides	4.9	g__Faecalibacterium	4.9	g__Barnesiella	4.4
Dampness	g__Veillonella	3.4	g__Paraprevotella	2.9	g__Clostridia_UCG-014	2.4
Qi deficiency	g__Alistipes	2.4	g__Bacteroides	2.4	g__Barnesiella	2.4
Phlegm	g__Bifidobacterium	1.0	g__un_f_Lachnospiraceae	1.0	g__Akkermansia	0.5
Heat	g__Bacteroides	0.5	g__Escherichia-Shigella	0.5	g__Faecalibacterium	−0.5
Heart	g__un_f_Enterobacteriaceae	1.5	g__un_f_Lachnospiraceae	0.5	—	—

Values are model-based feature importance weights expressed as percentages. Genera are ranked 1–3 per syndrome element. Negative values indicate a negative association with predicted severity. These metrics reflect variable importance for classification, not biological causality. un_f, unclassified family; —, not applicable.

Spleen: Supported by the broadest repertoire (57 genera), led by SCFA-producers Bacteroides(4.9%), Faecalibacterium(4.9%), and Barnesiella(4.4%). This aligns with the TCM concept of the spleen governing nutrient transformation.

Dampness: Dominated by Veillonella(3.4%) and Paraprevotella(2.9%). Veillonella’s role in lactate metabolism provides a plausible biochemical substrate for the TCM concept of “turbid dampness.”

Phlegm: Characterized by Bifidobacterium(1.0%) and unclassified Lachnospiraceae (1.0%). The diffuse nature of these signals suggests phlegm arises from broad ecological dysregulation rather than specific pathogens.

Heart: Driven by unclassified Enterobacteriaceae (1.5%), the primary source of LPS, linking gut dysbiosis to cardiovascular complications in T2DM.

Qi Deficiency: Distributed across Alistipes(2.4%), Bacteroides(2.4%), and Roseburia(1.8%), suggesting a systemic rather than taxon-specific association.

Heat: Marked by concurrent enrichment of pro-inflammatory taxa (Bacteroides, *Escherichia-Shigella*) and depletion of anti-inflammatory Faecalibacterium.

#### 3.6.2 SHAP interpretation

SHAP analysis provided individualized interpretability (**Figures 5B–D**). For spleen, Bacteroidesand Faecalibacteriumexerted the widest influence. Dependence plots revealed non-linear dose responses: Bacteroidesexhibited an optimal abundance window (~15–20%), beyond which marginal gains diminished. For dampness, Veillonellashowed a pathogenic threshold (~2% relative abundance), below which it had negligible impact. For phlegm, Bifidobacteriumdisplayed a biphasic curve; moderate abundance correlated with severity, whereas extreme abundance suggested ecological instability, consistent with a “dysbiotic” rather than “beneficial” role in advanced T2DM.

### 3.7 FMT clinical validation

Thirty-four patients completed the FMT intervention (20 T2DM, 14 T2DM with complications), receiving 20 capsules per session, once every other day, for three sessions (>2.5 × 10¹² CFU live bacteria per session).

#### 3.7.1 Biochemical response

Pooled analysis of the 34 recipients revealed significant improvements in glycemic control, β-cell function, and body composition following FMT (Wilcoxon signed-rank test; Benjamini–Hochberg FDR correction; **Supplementary Table S4**). HbA1c decreased from 7.30% (IQR: 6.93–8.28) to 6.50% (IQR: 5.95–6.88; q= 0.0006, r= −0.840), while both fasting and postprandial C-peptide levels rose significantly (q≤ 0.008, r≥ +0.630), indicating restored insulin secretory capacity. We also observed favorable anthropometric shifts, including reductions in BMI (26.17 to 25.21 kg/m²; q= 0.028, r= −0.533) and waist circumference (91.0 to 86.5 cm; q= 0.008, r= −0.669). All parameters surviving FDR correction exhibited large effect sizes (|r| ≥ 0.5).

Stratification by disease status revealed a marked disparity in treatment response. The T2DM subgroup (n= 20) drove the majority of these gains, with pronounced reductions in HbA1c (7.65% to 6.50%; q= 0.008, r= −0.914) and visceral adiposity (q≤ 0.050, |r| ≥ 0.68). Conversely, while the complications subgroup (n= 14) showed directionally consistent improvements—including a median waist circumference reduction of 12.5 cm—fewer parameters withstood FDR correction, likely owing to limited statistical power. Nonetheless, the large correlation coefficients observed for subcutaneous fat loss (r= −0.780) and ALT reduction (r= −0.838) suggest clinically meaningful metabolic remodeling even in this advanced phenotype.

#### 3.7.2 Microbial engraftment

In the T2DM group, 12 genera shifted significantly post-FMT (8 decreased, including Hungatella; 4 increased, including Sutterella), alongside 93 differential species. In the complications group, Senegalimassiliaincreased, while Anaerotruncusand Gordonibacterdecreased. Pooling cohorts, 15 genera and 106 species changed significantly (**Figure 6D**).

**Figure 6:**
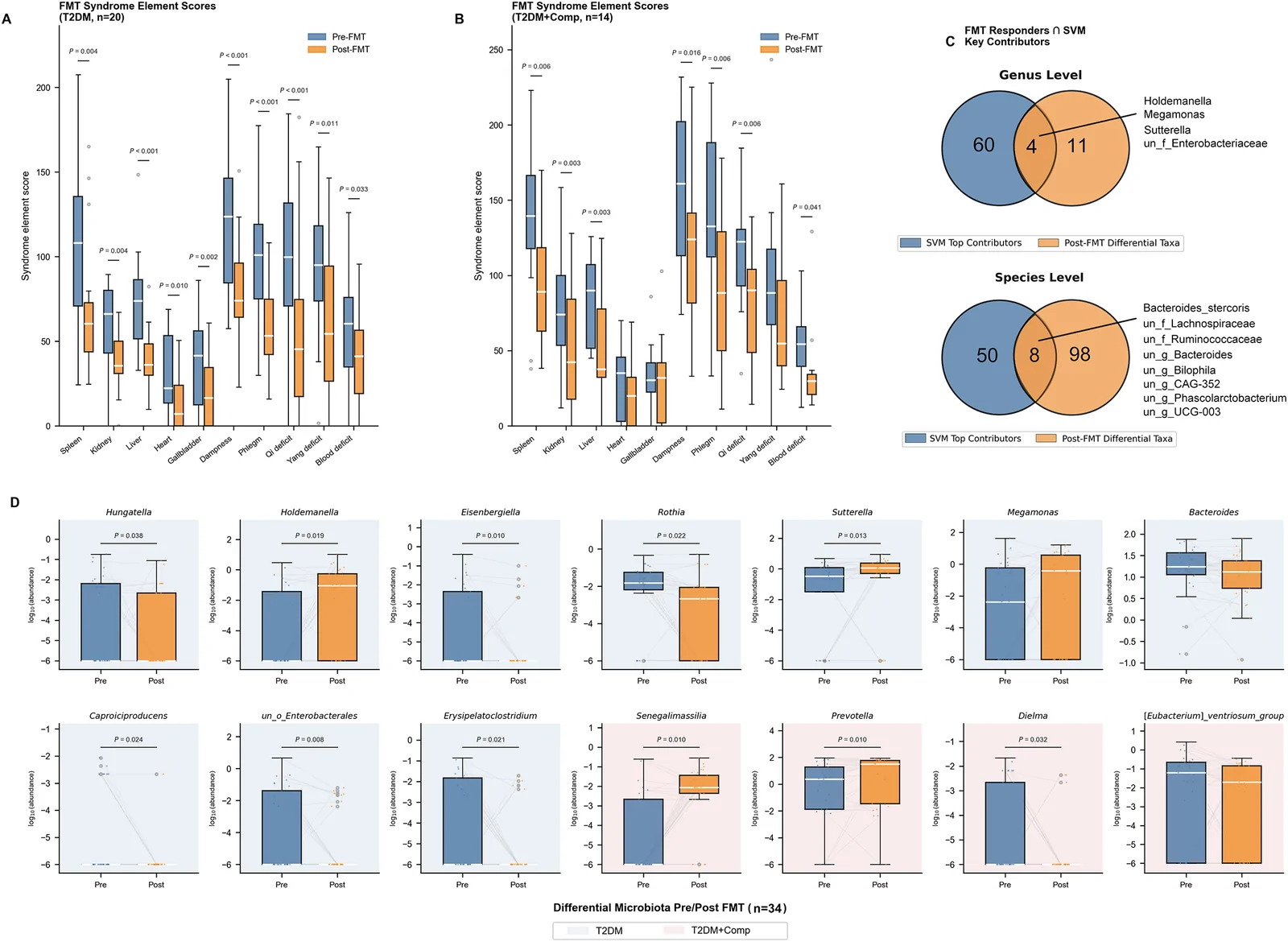
Validation of microbiota-syndrome links in an independent FMT cohort. **(A)** Changes in syndrome element scores pre- and post-FMT in T2DM recipients (n = 20; Wilcoxon signed-rank test). **(B)** Changes in syndrome element scores pre- and post-FMT in T2DM+Comp recipients (n = 14). **(C)** Venn diagrams showing overlap between post-FMT differentially abundant microbiota and top-ranking SVM features. Genus level: 4 overlapping genera out of 15 FMT-differential genera (pooled across both strata) and 64 SVM-contributing genera (26.7%). Species level: 8 overlapping species out of 106 FMT-differential and 50 SVM-contributing species (7.5%). **(D)** Paired abundance changes of differential genera pre-/post-FMT (n = 34), stratified by T2DM (blue panel) and T2DM+Comp (pink panel). Wilcoxon signed-rank test with Benjamini–Hochberg FDR correction.

#### 3.7.3 Syndrome element response

Responses were stratum-specific. The T2DM group showed broad reductions across organ (liver, kidney, spleen) and nature (qi deficiency, dampness, phlegm) elements. The complications group showed narrower improvements, primarily in kidney, spleen, and qi deficiency (**Figures 6A, B**).

#### 3.7.4 Clinical status transitions

FMT induced significant state shifts in T2DM patients: pre-FMT, 17 were diseased and 3 pre-diseased; post-FMT, 10 transitioned to pre-disease and 1 to non-diseased (*P* < 0.05). In the complications cohort, only 3 of 14 transitioned out of the diseased state.

#### 3.7.5 Model validation

We observed a 26.7% overlap (4/15 genera) between FMT-engrafted taxa and top SVM features (**Figure 6C**). Key taxa linked to spleen (*Bacteroides*, *Faecalibacterium*) and dampness (*Veillonella*) exhibited reciprocal shifts that correlated with syndrome score improvements (r= 0.42–0.61, *P* < 0.01). This alignment between computational prediction and biological intervention provides preliminary, yet convergent, support for the feature importance model.

## 4 Discussion and conclusions

### 4.1 Principal findings

This study provides the first quantitative, machine learning–based mapping of gut microbiota feature importance to individual TCM syndrome elements across the T2DM clinical continuum. Three principal findings emerged. First, T2DM progression follows a stepwise evolution in syndrome elements, transitioning from dampness, qi deficiency, and spleen involvement in pre-diabetes, through dampness, spleen, and phlegm in established T2DM, to dampness, phlegm, heat, and kidney involvement in complicated disease. This trajectory mirrors underlying shifts in microbiota composition. Second, SVM-based permutation importance and SHAP analysis identified syndrome element–specific microbial signatures, with *Bacteroides* and *Faecalibacterium* exhibiting the highest predictive weight for spleen, Veillonella for dampness, and *Enterobacteriaceae* for heart. Third, FMT-induced improvements in both metabolic parameters and syndrome element scores were directionally concordant with model predictions, evidenced by a 26.7% overlap at the genus level (4/15) between post-FMT differentially abundant taxa and top model contributors. These findings should be interpreted as hypothesis-generating, constrained by the cross-sectional design, modest sample sizes, and the inherently correlational nature of ML-derived feature importance.

### 4.2 Syndrome element–microbiota associations and biological plausibility

The high predictive importance of *Bacteroides* and *Faecalibacterium* for the spleen syndrome element is mechanistically compelling. Both genera are prolific SCFA producers; SCFAs activate GPR43 on enteroendocrine L-cells, stimulating GLP-1 and PYY secretion, thereby enhancing insulin sensitivity and glucose homeostasis. In TCM, the spleen governs the transportation and transformation of nutrients, a concept with clear parallels to digestive-absorptive physiology (Scheiman et al., 2019; Leite et al., 2022). The decline in *Faecalibacterium* abundance with T2DM progression, coupled with its high model weight for spleen, supports the hypothesis that SCFA production represents a biochemical correlate of spleen function.

The prominence of Veillonellain the dampness model warrants specific attention. Veillonellam etabolizes lactate to propionate via the methylmalonyl-CoA pathway (Tolhurst et al., 2012; Louis and Flint, 2017). While propionate confers benefits at physiological concentrations, excessive abundance in T2DM may signify disrupted lactate clearance. In TCM, dampness is conceptualized as the pathological accumulation of turbid metabolic products; thus, dysregulated organic acid metabolism provides a plausible biochemical substrate for this syndrome element.

Conversely, the positive association between *Bifidobacterium* and phlegm severity presents an apparent paradox, given the genus’s established probiotic reputation. However, the endogenous over representation of *Bifidobacteriumin* T2DM may reflect broader ecological dysbiosis, such as the high-carbohydrate “*Bifidobacterium*-dominant” enterotype (Wu et al., 2011). SHAP analysis clarified this relationship, revealing a biphasic dose-response where predictive importance plateaued and reversed at very high abundances. This underscores the necessity of quantitative, context-dependent assessment over binary classifications of taxa as merely “beneficial” or “detrimental.”

Finally, the association between *Enterobacteriaceae* and the heart element aligns with the pathophysiology of T2DM complications. *Enterobacteriaceae* are the primary source of circulating LPS, driving TLR4-mediated inflammatory cascades that contribute to cardiovascular complications (Cani et al., 2007; Creely et al., 2007; Hotamisligil, 2017; Chen et al., 2011). This finding resonates with the TCM understanding of heart involvement in advanced disease, particularly concerning diabetic cardiovascular autonomic neuropathy (Vinik and Erbas, 2013).

Prior studies consistently demonstrate that TCM interventions exert therapeutic effects via gut microbiota remodeling, with meta-analytic evidence supporting benefits for lipid metabolism disorders (Gong et al., 2022) and empirical data showing restoration of microbial diversity in antibiotic-associated diarrhea (Hui et al., 2020).These regulatory effects extend beyond localized gastrointestinal outcomes to systemic pathways, including the gut-heart axis engaged by Jiashen Prescription in heart failure (Cui et al., 2023) and the renal-intestinal axis proposed as a mechanistic basis for TCM fluid metabolism regulation (Yu et al., 2025).Building on these established associations, our study advances the field by quantitatively mapping specific gut taxa to discrete TCM syndrome elements, refining traditional holistic assessments into granular, microbiota-informed diagnostic frameworks.

### 4.3 Three-stage syndrome evolution and microbial dynamics

The observed progression of syndrome elements parallels the natural history of T2DM pathogenesis (Tabák et al., 2012). In pre-diabetes, insulin resistance corresponds to TCM dampness and spleen dysfunction. As beta-cell function deteriorates, metabolic byproducts accumulate, reflecting the TCM concept of dampness congealing into phlegm. The emergence of heat and kidney involvement in the complication stage signifies systemic inflammation and end-organ damage, particularly diabetic nephropathy.

These clinical stages correspond to distinct microbial dynamics. Pre-diabetes is characterized by moderate reductions in SCFA producers. Established T2DM involves a further loss of butyrate producers and the emergence of *Veillonella*. The complication stage features a dominance of proteolytic bacteria and increased LPS burden. Our feature importance model captures these stage-specific associations quantitatively, providing a molecular framework for what might be termed “micro-syndrome differentiation.”

### 4.4 Integration of MR, ML, and FMT: a multi-evidence approach

The three analytical components constitute complementary lines of evidence rather than identical replicates. Mendelian randomization served to identify taxa causally linked to T2DM at the genetic level, providing biological a prioricandidates. Notably, 11 of the 21 MR-identified genera (52.4%) from East Asian cohorts overlapped with top SVM features, including *Prevotella*(spleen) and *Enterobacteriaceae* (heart). However, the absence of GWAS data for TCM syndrome elements precludes direct MR analysis of these specific phenotypes.

The logical progression of this study integrates these components: MR identifies microbial candidates anchored in T2DM pathophysiology; ML maps these candidates onto specific syndrome element profiles; and FMT provides an interventional perturbation. The 26.7% overlap between post-FMT engrafted taxa and SVM features, alongside significant correlations between microbial shifts and symptom improvements (r= 0.42–0.61), offers convergent preliminary support for the model. It is crucial to recognize that these components address different levels of evidence—genetic liability, observational correlation, and interventional response. Their integration provides a robust hypothesis-generating framework rather than definitive confirmation of causality.

### 4.5 Methodological considerations

Several methodological features distinguish this work. The syndrome element system offers granularity beyond holistic TCM diagnoses, decomposing phenotypes into location and nature axes. The nested cross-validation framework provides an unbiased estimate of model performance, mitigating optimism bias. While absolute classification performance (AUC 0.44–0.76) was modest, this reflects an honest quantification of the information ceiling of 16S rRNA data for complex phenotypes, rather than a failure of the modeling approach.

Of particular interest, the Kidney (Shen) element was the only syndrome element to show consistent significance across all advanced metrics at both taxonomic levels (genus ρ = 0.18, *P* = 0.02; species ρ = 0.23, *P* = 0.01). This aligns with the TCM tenet of the Kidney as the “congenital foundation” governing systemic metabolic homeostasis. The significant Spearman correlation, despite its modest magnitude, indicates that 16S data carries consistent directional information for this specific element, whereas others may require deeper omics resolution.

### 4.6 Limitations

Several limitations warrant acknowledgment. The single-center design and modest sample sizes constrain generalizability (Gupta et al., 2017). The cross-sectional nature of the main cohort precludes causal inference regarding temporal relationships. While 16S sequencing is cost-effective, it lacks the functional resolution of metagenomics; future studies should integrate shotgun sequencing to resolve strain-level variations and metabolic pathways.

TCM syndrome assessment, despite standardization, retains subjective elements. While two practitioners evaluated participants independently, formal inter-rater reliability statistics (e.g., ICC) were not systematically calculated, which represents a limitation. Furthermore, the FMT cohort was small (n= 34) and lacked a placebo control, limiting causal inference from the interventional arm. *Post-hoc* power analysis suggests we were adequately powered to detect large effects (d > 0.7), but potentially underpowered for subtle microbial shifts.

Residual confounding from pharmacotherapy remains a concern. Metformin, prescribed to the majority of T2DM patients, exerts well-documented effects on the gut microbiome (e.g., enriching *Escherichia-Shigellaand Akkermansia*). This creates a structural imbalance, as pre-diabetic participants were largely drug-naïve. We attempted to address this via stratified analysis in metformin non-users and partial correlation adjustments (**Supplementary Table S3**); however, the restricted sample size in these sub-analyses limits statistical power. Dietary patterns, a major confounder, were not formally assessed via food frequency questionnaires in either cohort. Consequently, the observed microbiota–syndrome associations should be viewed as exploratory correlations rather than evidence of direct causality.

### 4.7 Clinical implications and future directions

The microbiota–syndrome element atlas offers translational potential. It provides quantitative biomarkers to supplement traditional diagnostics, such as the *Bacteroides/Faecalibacteriumratio* for spleen health. Theoretically, these profiles could guide personalized FMT donor matching or the design of targeted synbiotic interventions (Kijmanawat et al., 2019). However, these applications remain speculative pending validation.

Future research must prioritize multi-center prospective cohorts with standardized assessments. Randomized controlled FMT trials, stratified by baseline syndrome element profiles, are necessary to test whether model-guided interventions improve clinical endpoints. Integrating host genomics, metabolomics, and dietary data will be essential to refine these predictive models.

## 5 Conclusions

In conclusion, this study establishes a quantitative framework linking gut microbiota composition to TCM syndrome elements in T2DM. We demonstrate that the progression of syndrome elements across disease stages is paralleled by specific microbial shifts. By integrating Mendelian randomization, machine learning, and FMT intervention, we provide convergent evidence supporting a quantitative model of microbiota–syndrome interactions. This work bridges TCM diagnostic theory with modern microbiome science, laying the groundwork for micro-syndrome differentiation and personalized microbiota-targeted therapies in metabolic diseases. Definitive confirmation of causality awaits larger, controlled interventional studies.

## Data Availability

The original contributions presented in the study are included in the article/**Supplementary Material**. Further inquiries can be directed to the corresponding author/s. Custom Python and R scripts for SVM modeling, permutation importance, SHAP analysis, and MR analysis are available from the corresponding author upon reasonable request.
