# Supplementary material for: Quantitative mapping of gut microbiota feature importance to TCM syndrome elements in clinical T2DM stages
Source: Front Cell Infect Microbiol. 2026 Sep 24;16:1910757. doi: 10.3389/fcimb.2026.1910757 (PMC13645698; doi:10.3389/fcimb.2026.1910757)
Supplement: Supplementary file 1 [file DataSheet1.pdf]

Supplementary Material

Supplementary Figures

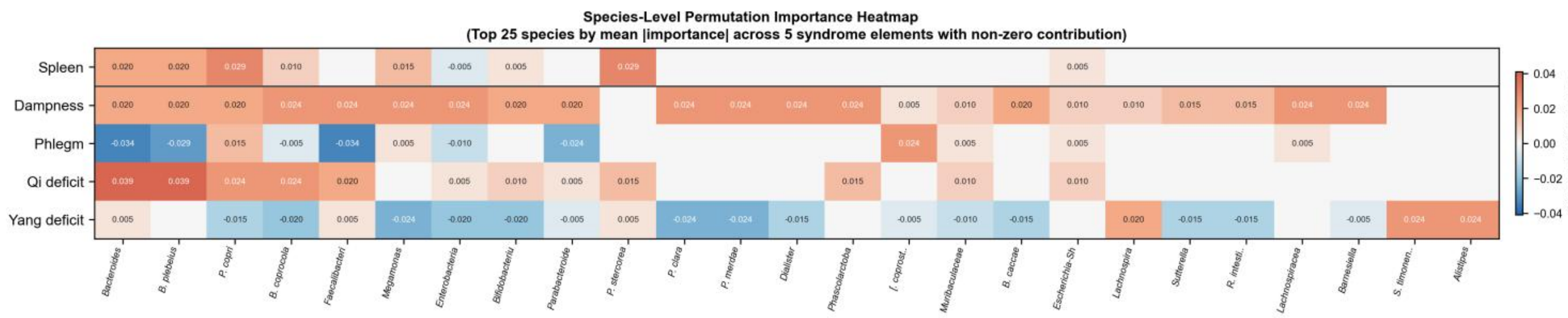

Supplementary Fig. S1. Species-level permutation importance heatmap. Top 25 species ranked by mean permutation importance across the five TCM syndrome elements exhibiting non-zero feature contributions: *Kidney (Shen)*, *Qi stagnation*, *Yin deficiency*, *Heat*, and *Phlegm*. Values represent permutation importance scores, computed as the mean decrease in SVM classification AUC when each feature column is randomly shuffled 30 times (Altmann et al., 2010) within a nested 10-fold cross-validation framework (repeated 5 times). These scores reflect predictive utility for syndrome element classification and should not be interpreted as direct biological contribution percentages. Importance weights were normalized to a 0–1 scale within each element model. Red indicates positive association (higher species abundance predicts greater syndrome severity); blue indicates negative association (higher abundance predicts lower severity). Empirical significance was derived from 99-permutation testing: *$P < 0.05$; **$P < 0.01$. The analysis was performed on 201 participants with paired metagenomic sequencing and TCM syndrome element evaluation.

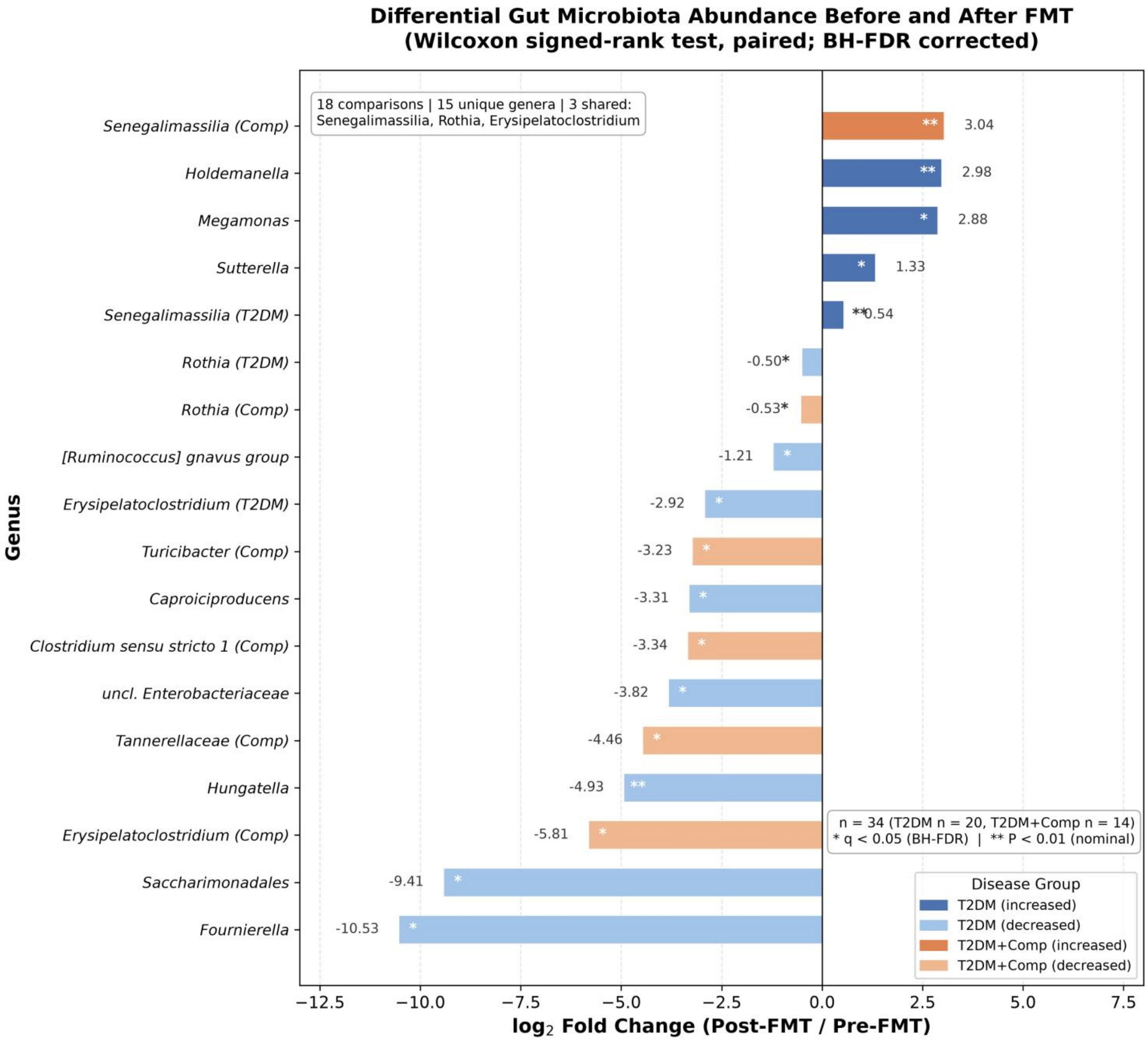

Supplementary Fig. S2. Differential gut microbiota abundance before and after FMT. Horizontal bar plot showing the log2-fold change (post/pre FMT) for 15 genera with nominal P< 0.05 (Wilcoxon signed-rank test, paired; n= 34 total; T2DM, n= 20; T2DM with complications, n= 14). Analyses were stratified by disease status; three genera (*Senegalimassilia*, *Rothia*, and *Erysipelatoclostridium*) were significant in both strata. Benjamini–Hochberg FDR-corrected q-values are reported alongside nominal P-values; all 18 comparisons retained significance at q< 0.05 (range: q= 0.035–0.048). Red bars indicate enrichment post-FMT; blue bars indicate depletion.

Intersection of MR-Causal Microbiota and ML Feature Importance

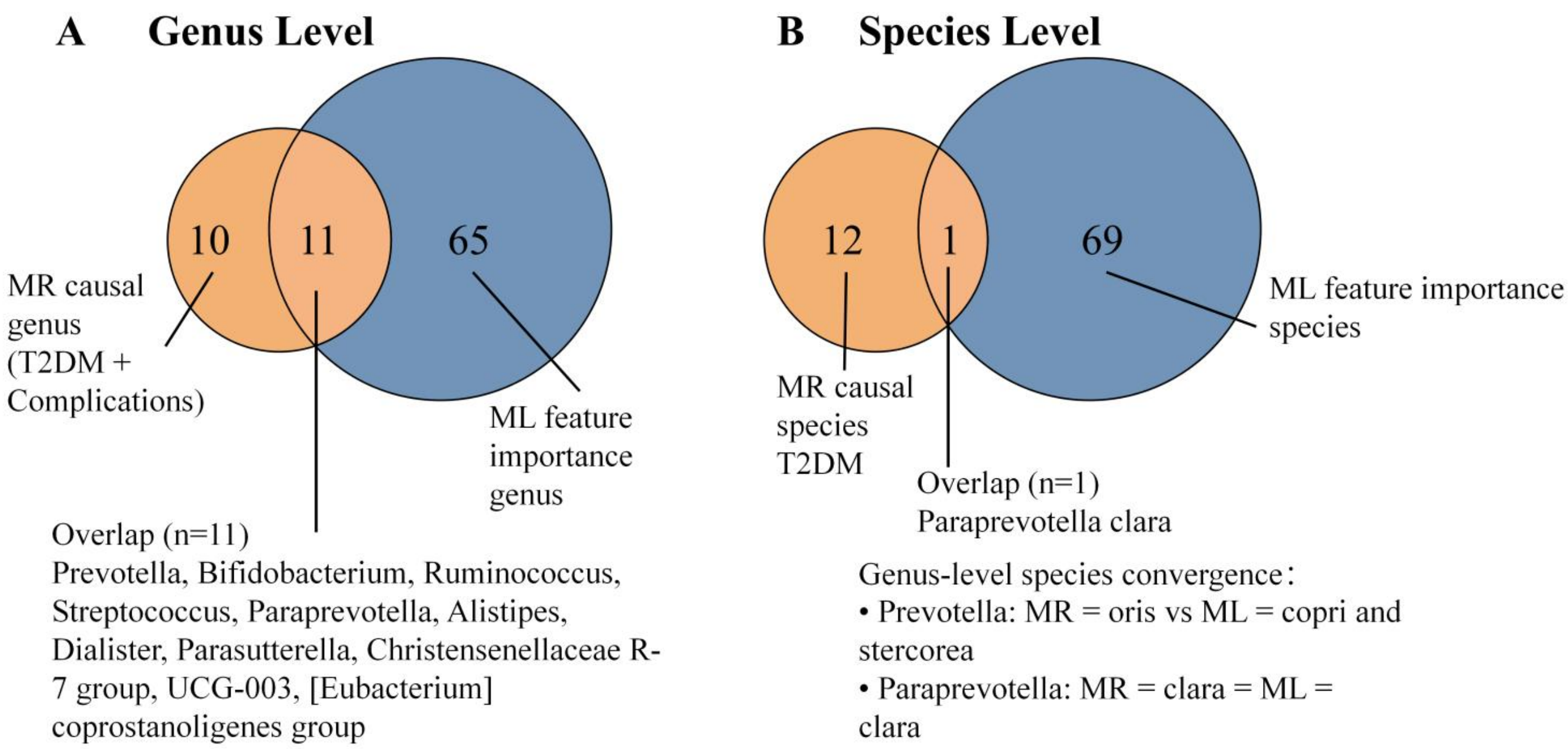

Supplementary Fig. S3. Intersection of Mendelian randomization (MR) – causal gut microbiota and machine learning (ML) feature importance. (A) Genus-level overlap between MR-causal genera (combined T2DM and T2DM-with-complications phenotypes, n= 21) and top ML feature-importance genera (n= 76). Eleven genera overlapped: *Prevotella*, *Bifidobacterium*, *Ruminococcus*, *Streptococcus*, *Paraprevotella*, *Alistipes, Dialister, Parasutterella, ChristensenellaceaeR-7 group, UCG-003, and [Eubacterium] coprostanoligenes group*. (B) Species-level overlap between MR-causal T2DM species (n= 13) and top ML feature-importance species (n= 70). Only Paraprevotella clarawas shared. Genus-level congruence is annotated for *Prevotella*(MR-causal: *Prevotella oris*; ML feature-importance: *Prevotella copri* and *Prevotella stercorea*) and *Paraprevotella*(shared species: *Paraprevotella clara*). MR, Mendelian randomization; ML, machine learning; T2DM, type 2 diabetes mellitus.
